# Ectropion in A Case of Collodion Baby

**Published:** 2014-07

**Authors:** Nikhil Panse, Parag Sahasrabudhe

**Affiliations:** Department of Plastic Surgery, BJ Medical College and Sasoon Hospital, Pune, India

## DEAR EDITOR,

We had the opportunity to manage a case of bilateral ectropion in a collodion baby. Collodion baby is a rare congenital disorder characterized by parchment like taught membrane covering the whole body. Incidence of this condition is 1 in 300,000 live births.^1^

**Fig. 1:**
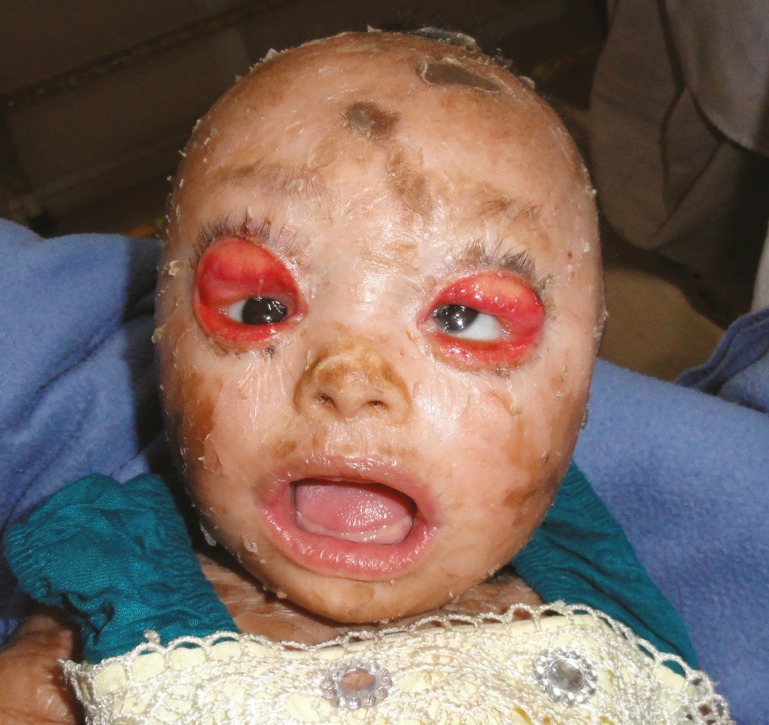
Ectropion in a three months old collodion baby.

A three months old collodion baby was referred to us for management of bilateral ectropion. An appropriate-for-gestational-age boy was born at 39 weeks’ gestation by a normal vaginal delivery of a 27-year- female, with an unremarkable pregnancy and of a non-consangious marriage (Figure 1).

On examination, the patient had bilateral ectropion of both the upper and lower eyelids. The eyelashes on the lower eyelids were missing. There was no evidence of corneal opacities or keratitis. The ears were small, low set and underdeveloped. The nose was flattened. There was slight eclabium, and the mouth was constantly in an open position and like a fish. The whole body was covered with parchment-like membrane resembling collodion and was peeling off on the entire body including the face. Skin biopsy was done elsewhere and showed findings suggestive of collodion baby with epidermal hyperkeratosis and preservation of granular layer.

Collodion baby be a manifestation of various conditions,^1^ but often it is a manifestation of lamellar ichthyosis. Collodion baby represents difficult treatment challenge, because of prematurity, dehydration, temperature instability, and infection.^1^

When the baby was referred to us for management of ectropion, it was hemodynamically stable, and there was no evidence of any infection, dehydration or compromised vitals. There was no evidence of any corneal involvement. Most often ectropion with corneal exposure is managed on an immediate basis for the fear of developing corneal injuries, opacities and keratitis. Ectropion is generally managed by release and coverage either by a thick split thickness skin graft or a full thickness skin graft. 

This baby presented with this unique problem, wherein the quality of skin was compromised. There was a parchment like layer over the skin with constant peeling. There is evidence which suggested that ectropion in colloidon babies can be managed conservatively by local application of clobetasol in older children.^2^

We managed the patient conservatively by the use of clobetasol for local application over the eyelids and regular use of lubricants in the eye. Emollients were advised for skin softening and moistening. Patient was advised weekly follow up to a local ophthalmologist to identify the slightest corneal involvement. 

There is not enough literature on collodion babies, healing potential of their skin, surgical intervention and their long term follow up. Although we have managed the ectropion conservatively, we intend to go in for surgical intervention at the first indication of any corneal involvement. More studies and reports, especially on the management of surgical aspects of collodion babies are needed to help us manage these patients in a better way. 

## CONFLICT OF INTEREST

The authors declare no conflict of interest. 

## Footenote

Please cite this paper as:

Mohammadi AA, Seyed Jafari SM. Trichoepithelioma: A Rare but Crucial Dermatologic Issue. World J Plast Surg 2014;3(2):146-147.
